# Bariatric psychology in the UK National Health Service: input across the patient pathway

**DOI:** 10.1186/s40608-014-0020-6

**Published:** 2014-08-30

**Authors:** Denise Ratcliffe, Rukshana Ali, Nell Ellison, Mahbuba Khatun, Jolyon Poole, Caroline Coffey

**Affiliations:** Psychological Medicine Unit, CNWL NHS Foundation Trust, 1 Nightingale Place, London, SW10 9NG UK; Bariatric Surgery Service, Chelsea & Westminster NHS Foundation Trust, London, UK; Department of Clinical, Educational & Health Psychology, University College London, London, UK; Phoenix Health, Chester, UK

**Keywords:** Bariatric surgery, Psychology, NHS, Provision

## Abstract

**Background:**

Providers of bariatric surgery within the National Health Service (NHS) are required to provide psychological assessment and intervention, yet operational definitions regarding the purpose and scope of this input are lacking. This has led to significant variation in the provision of psychology, with some providing an assessment-only service and others providing a more comprehensive package of intervention throughout the patient pathway. The aims of this paper are to document the current psychology provision and service models of National Health Service (NHS) bariatric surgery services in the UK.

Psychologists belonging to a bariatric psychology forum completed a survey. This focused on provision of psychological assessment and intervention throughout the bariatric pathway as well as the ratio between psychology resources and number of bariatric procedures per year. We obtained information from 22 NHS services which provide 3691 procedures per year.

**Results:**

There is significant variation in the ratio between psychology resources and number of bariatric procedures undertaken per service. Whilst all services offer pre-surgery psychology assessments, less than one-third routinely assess all potential bariatric surgery candidates. Over 90% of services offer pre-surgery individual interventions and 41% offer pre-surgery groups. None of the services routinely offer post-surgery assessments but 68% offer post-surgery assessment and intervention following referral. None offered post-operative structured psychological group interventions.

**Conclusion:**

There are significant disparities and inconsistencies in the provision of psychology resources in relation to surgery volume in the NHS. Most of these resources are directed at pre-surgery assessment and this raises issues regarding the function of these assessments. Rather than focusing on assessing psychological (un)suitability for surgery, an evidence-based approach involves psychologists offering pre-operative interventions to improve readiness for surgery and post-operative interventions to address recurring or emerging difficulties which impact on outcomes.

## Background

The rates of bariatric surgery have increased exponentially in the UK in the last decade [1]. Individuals seeking bariatric surgery in the UK may access this treatment through the National Health Service (NHS) if they meet eligibility criteria outlined by the National Institute for Clinical Excellence (NICE) and NHS Commissioning Board for Complex and Specialised Obesity Surgery. Alternatively, some individuals privately fund their bariatric surgery though independent healthcare organisations. Although providers of bariatric surgery within the NHS are required to provide psychology, operational definitions regarding the purpose and scope of this are lacking. This means there is significant variation in the provision of psychological input across bariatric services, with some providing an assessment-only service and others providing a more comprehensive package of intervention throughout the patient pathway. This paper aims to document the current psychology provision and service models of NHS bariatric surgery services in the UK. The paper focuses on the distribution of psychological resources and input across the bariatric pathway i.e. how much time a psychologist has within a service on a weekly basis, and where the provision is focused e.g. pre-operative, post-operative or both, rather than the specific methods or modalities of assessment and intervention (e.g. cognitive behavioural therapy). The NHS is a taxpayer funded healthcare system, which is free at the point of need, although there are geographical variations in resources which are likely to lead to differing service models/provision of care across the UK as well as in comparison to other countries. Given the significant range and complexity of psychological issues that may impact on, and arise from, bariatric surgery it is important to identify the provision, and variation in psychological assessment and intervention across a bariatric pathway in order to gauge how these might be identified and addressed. We therefore start by summarising the major psychological issues that arise pre and post-surgery before moving on to review the literature on bariatric psychology services elsewhere.

### Psychological difficulties across a bariatric surgery pathway

These can be roughly categorised as a) pre-existing psychological difficulties, b) psychological difficulties specifically related to the behaviour changes involved in bariatric surgery and c) post-operative development of new difficulties or reactivation of pre-existing difficulties.

Rates of anxiety, depression [2], personality disorders [3], physical, sexual and emotional abuse [4] and suicide attempts [5] are higher in bariatric surgery candidates compared to the general population. However, no consistent pre-operative psychiatric predictors of weight loss outcomes have been identified [6]. Prevalence rates for binge eating disorder (BED) vary significantly but on average, approximately one-quarter of bariatric surgery candidates meet diagnostic criteria [7]. BED is not a formally recognised contraindication for surgery even though it has been found to contribute to less weight loss and weight regain [8]. This subgroup requires active monitoring and may require both pre and post-operative interventions to attenuate the risk of poor outcomes. Regardless of pre-operative mental health vulnerabilities, bariatric surgery requires major changes to eating behaviour and development of new skills, which given the individual’s weight and dieting history, may be uncongenial or difficult to maintain.

In terms of post-operative psychological difficulties, these divide into those which are rare but potentially catastrophic, and subclinical difficulties associated with adjustment to major changes in the individual’s weight, shape and identity. BED (or loss of control eating) is the most common eating disorder to re-emerge following bariatric surgery and is predictive of poorer weight loss at 12 months and 24 months [8]. Conversely, a subgroup of bariatric patients develop an anorexic/bulimic eating profile which is associated with greater than expected weight loss and/or purging [9]. There is emerging evidence of increased alcohol intake in a subgroup of individuals who have gastric bypasses [10]. There have been reports of increased suicide rates amongst those who have had bariatric surgery compared to the general population [11] with 70% of these suicides occurring during the first three years following surgery [12]. In contrast to the psychological presentations highlighted so far, most psychological difficulties that individuals experience following surgery are not attributable to diagnosable mental health problems, and are a consequence of undergoing a major life/body change. A recent survey [13] found that coping with excess skin and body image issues were the most problematic issues experienced by post-operative patients. Other difficulties included maintaining eating behaviour changes, emotional eating and unmet weight loss expectations.

### Service models of psychology provision

Most of the literature on bariatric psychology practice has historically focused on pre-surgery psychological assessments and a number of studies have highlighted the lack of standardisation and wide variation in assessment methods, outcome decisions and recommendations [14,15]. Previously the motivation for such assessments has been the identification of psychological factors to identify suitable (and unsuitable) candidates and enable better prediction of weight loss outcomes. However, clear psychological/psychiatric contraindications to bariatric surgery have not been identified and studies have revealed varying practice in terms of decisions about postponing or declining bariatric surgery on these grounds [16]. More recently, it has been argued that the function of the pre-operative psychological assessment is not to “gatekeep” access to bariatric surgery and it has evolved towards identifying psychosocial challenges and risk factors which may jeopardise weight loss and psychosocial outcomes for the individual [17]. These assessments focus on readiness/preparedness for surgery with individuals viewed as being on a continuum rather than the dichotomous outcomes generated from a suitability assessment [18]. With regard to frequency of pre-surgery psychological assessments, Bauchowitz et al. [14] reported that almost 90% of the 188 bariatric programs surveyed in the USA required all candidates to have a psychological assessment [14]. Other studies have reported that the frequency of psychological assessments for bariatric candidates can range between 67% and 92% (for Holland and USA respectively) [19,20].

Most of the literature documenting bariatric psychology practice focuses on the methods, outcomes and frequency of psychological assessments and there is limited information regarding service provision beyond this. However, in the U.S., there are guidelines [21] which make specific recommendations about psychological services, for example that mental health resources should be available beyond the 6 month post-operative period in order to address emerging difficulties or complications. Patients and provider’s compliance with these recommendations is unclear as a survey found that 57% of bariatric patients who completed a survey in the U.S. did not have any psychology follow up appointments in the 12 months following surgery [22]. The type of healthcare system is also likely to impact on service provision as healthcare insurance providers may stipulate specific requirements for psychological input. Whilst guidelines exist within the UK [23,24], these simply state that psychologists should be part of the multidisciplinary team rather than outlining specific requirements of the role. Consequently, there is a lack of information about how bariatric psychology services operate within the NHS and we therefore conducted a survey of psychologists working within these services.

## Methods

57 psychologists belonging to a UK obesity/bariatric psychology online forum hosted by the British Psychological Society (BPS) were asked to complete a survey which was emailed back to the authors (DR). Membership of this forum consists of psychologists who work in bariatric surgery and/or weight management services. It is not possible to ascertain how many of the 57 psychologists who were sent the survey work in bariatric surgery compared to non-surgical weight management services. Respondents were asked to a) provide information on the ratio of psychology input to number of patients and the type of team that the psychologist worked in b) the scope of the psychology service throughout the bariatric pathway. We intentionally restricted our sample to NHS providers of bariatric surgery as opposed to private healthcare providers in order to keep the organisational and financial framework standardised. The NHS Research Ethics Authority guidelines and online review tool indicated that this survey of psychologists does not fulfil criteria for formal research and therefore ethical review by an NHS Ethics Committee was not required.

## Results

We obtained data from psychologists working in 22 NHS bariatric surgery services and this represents 39% of the psychologists belonging to the BPS online forum. We used data from the National Bariatric Surgery Register (NBSR) [25] to identify 43 NHS bariatric surgery units (it is possible that this is an underestimate as this is a voluntary register) which means we have survey responses from 51% of these bariatric units.

### Part A: surgery volume and psychology resources

Table 1 contains data from 22 UK services (each represented by a letter to retain anonymity) and shows the number of bariatric procedures per year alongside the amount (WTE - whole time equivalent) of psychology time available. Whole Time Equivalent (WTE) is a standardised method for describing the amount of psychology time available per week with reference to standard NHS working hours (37.5) as the denominator. For example, if a service has more than one full-time psychologist, the WTE will be greater than 1.0 or if the psychologist works 2.5 days per week (i.e. 18.75 hours), they will have a WTE of 0.5.

**Table 1:** **Data provided by 21 bariatric services on the number of bariatric procedures performed and the amount of psychology resources available by site**

**Service**	**Number of procedures per year**	**Psychology time available per week** ^**a**^
		**(0.1 = half a day per week, 1.0 = 5 days per week)**
**0-100 procedures**		
A	5	0.2
B	40	.5
C	45	.2
D	48	.2
E	50	0.5
F	50	0.6
G	70	0.4
H	80	1.7
I	75	0.1
J	80	0.1
K	84	.3
L	Not sure	0.4
M	100	.25
**101-200 procedures**		
N	135	0.4
O	199	0.4
P	200	0.2
**201-500 procedures**		
Q	230	0.6
R	250	0.2
S	450	2.5
T	400	0.5
U	450	1.2
V	500	0.8

As discussed earlier Whole Time Equivalent (WTE) refers to the number of psychology hours available in a week using a denominator of the standard NHS fulltime working hours (37.5). A WTE of 0.1 equates to half a day per week, 1.0 equates to 5 days per week and greater than 1.0 indicates that there is more than one fulltime psychologist in the service (e.g. 1.2 WTE = 1 fulltime psychologist plus 1 day of another psychologist).

The total number of procedures across 21 (data not provided by one site) services is 3691. The NHS data available from 2012–2013 (which corresponds to when this survey was conducted) indicates that 8024 NHS bariatric procedures were undertaken in this year [26] and therefore the services included in this survey represent services which performed 46% of new bariatric procedures in the NHS.

The variation in the amount of available psychology time and number of procedures is striking. For example, service R which performs 250 procedures has the same amount of psychology input (.2 WTE) as service C which performs 45 procedures. Additionally, service S has more than twice as much psychology provision as similar high volume services U and V.

### Does the psychologist work as part of the bariatric multidisciplinary team?

Of the 22 services that provided data, 13(59%) psychologists worked within the bariatric team, 5(23%) were part of a specialist weight management service, 3(14%) worked in a clinical health psychology department and 1(4%) worked in an eating disorder service. The majority of respondents (91%) attended the bariatric multidisciplinary team meeting.

### Part B: psychological activity within bariatric surgery services in the NHS

Respondents provided data on the amount of psychology assessment and intervention they provided throughout the bariatric surgery pathway.

### Preoperative psychological input

#### Assessment

Participants were asked if their service offered pre-surgery psychology assessments and if so, whether this was routine for all patients. All 22 services indicated that they provide pre-surgery psychological assessments, however the majority of these services only assessed selected patients. Only seven services (32%) actually assessed all new patients (using a clinical interview), with the rest relying on triage by another member of the multidisciplinary team.

#### Interventions

Respondents were asked whether they provided individual or group psychological interventions prior to surgery. Twenty (91%) services reported that they provided individual interventions for pre-operative patients. These primarily focused on providing interventions for eating difficulties (e.g. binge eating, emotional eating, night eating) in order to facilitate preparation for surgery. Nine (41%) services indicated that they provide pre-surgery groups for patients and these varied widely from solo seminar sessions to 12 session programs.

### Post-operative psychology input

Fifteen (68%) services indicated that they have the capacity to offer post-surgery psychological assessment and intervention but this was not routinely provided. Individuals were referred by other members of the multidisciplinary team. Fifteen (68%) services offered post-surgery individual psychological interventions. Support groups were provided by some services but none reported that they were currently providing structured, focused therapy groups to address post-operative difficulties or adjustment.

### Psychologist’s view of their role and input

#### Psychologist as “gatekeeper” to surgery

Respondents were asked to indicate how frequently they thought that other members of the multidisciplinary team perceived the function of the pre-surgery psychological assessment as being to control access to bariatric surgery through “gatekeeping”. The gatekeeping role refers to whether others (team/patients) view the purpose of the psychological assessment as being the determination of suitability for surgery. Table 2 indicates that fifteen (68%) psychologists reported that they “sometimes” or “often” felt that they were perceived as the gatekeeper to bariatric surgery.

**Table 2:** **Responses to the perceived role of a psychologist in a bariatric surgery team**

**In your service, to what extent is the psychologist viewed as being a gatekeeper to bariatric surgery?**			
**Not at all**	**Rarely**	**Sometimes**	**Often**
3 (13.6%)	4 (18.2%)	12 (54.5%)	3 (13.6%)

#### Input through the pathway

Respondents were asked for their view of where psychological input was most needed across the bariatric pathway and the responses are shown in Table 3. The majority of respondents (63.6%) indicated that they felt both pre and post-operative psychological input was needed rather than prioritising pre-operative or post-operative input.

**Table 3:** **Views on where psychological input is most needed within the bariatric pathway**

**Where do you feel that psychology input is most needed within your service?**		
**Pre**	**Post**	**Both pre and post (i.e. throughout the pathway)**
18.2% (4)	18.2% (4)	63.6% (14)

## Discussion

This survey data reveals stark differences in the level of psychology provision across NHS bariatric services in the UK reflecting adhoc arrangements rather than systematic planning based on volume of patients and psychological need. Similarly to other surveys of service provision [20,22], most psychological resources appear to be focused on pre-operative assessment and intervention rather than post-operative input. However, evidence for prioritising in this way is lacking given the emergence of post-operative psychological difficulties which affect weight loss outcomes [8,10]. It could be argued that there is a disparity between where psychological resources are currently focused and where they are likely to be of most benefit.

The data indicates that whilst most services offer pre-surgery assessments, only one-third assess all bariatric surgery candidates. This is significantly different from the practice reported in other surveys [14,19,20] whereby the vast majority of bariatric candidates routinely had a psychological assessment. The majority of UK NHS bariatric services refer selected bariatric candidates for psychological assessment but this process raises the thorny issue of who should have a pre-surgery psychological assessment and in more general terms, the purpose of a psychological assessment prior to surgery. Interestingly, a significant proportion of respondents felt they were placed in the “gatekeeping” role, indicating the need for education about the role of the psychological assessment within bariatric multidisciplinary teams (especially as this view will impact on how patients subsequently engage and respond to psychologists). The perceived emphasis on the gatekeeping role suggests a different view of pre-operative psychological assessments to other countries, whereby the focus is on readiness for surgery. It is possible that this misperception of the function of the psychological assessment in the UK is reinforced by the rationing culture of the NHS where resources are limited. Pre-operative psychological assessments should be undertaken to identify and assist individuals with psychosocial problems that might impair their weight loss and psychosocial outcomes. Moreover, if assessments are viewed as an opportunity to identify challenges and readiness for bariatric surgery, this logically involves offering interventions to ameliorate these psychological risk factors. This is predicated on the basis that services are commissioned to provide such interventions and whilst this may be the case in other countries, the variation in resources in NHS services will undoubtedly have an impact on the capacity of these services to identify difficulties and provide evidence-based interventions.

The results from this survey indicated that a surprisingly low number of services (41%) provide pre-operative groups for bariatric candidates and this reflects a divergence from the evidence base. Pre-surgery groups increase patient’s preparedness and readiness for bariatric surgery and are associated with increased ability to implement and adhere to post-operative changes [27]. Patients attending a pre-operative behavioural lifestyle intervention, compared to usual care, lost significantly more weight prior to bariatric surgery [28]. Furthermore, a meta-analysis found that patients who lost weight prior to surgery had greater weight loss one year after surgery [29]. Ashton et al. [30] reported that individuals who were positive responders to a pre-operative binge eating group had better post-operative weight loss outcomes compared to non-responders [30].

Two-thirds of services offer post-operative assessments and follow up appointments but these are not routine for all patients. Therefore, the onus is on other members of the multidisciplinary team to detect and highlight psychological difficulties as they continue to see patients post-operatively and there is a lack of post-operative psychological follow up appointments. This approach is vulnerable to individual differences in level of skill, confidence and sensitivity and research demonstrates that health professionals are generally poor at detecting distress [31]. Whilst these data inform us about the potential of services to provide input, we are not able to determine the proportion of patients that actually received psychology follow up. Given the serious post-operative difficulties with binge eating and other eating disorders alongside the increase in alcohol misuse and suicide, it could be argued that it should be routine for all post-operative bariatric patients to have psychology follow upas this would enable early detection of emerging difficulties and rapid intervention. A U.S. survey reported that whilst less than 50% of post-operative bariatric patients had psychology follow up appointments in the first year after surgery, those patients who accessed these appointments had greater excess weight loss [22].

Whilst some of the services offered support groups, none provided structured post-operative intervention groups. A meta-analysis of post-operative groups and weight lossconcluded that psychological interventions alongside surgery are associated with better outcomes in terms of weight loss than bariatric surgery alone [32].Whilst a positive relationship has been found between support group attendance and weight loss [33], there are clear differences between support groups and structured psychological intervention groups addressing specific difficulties. There is a need for targeted groups to address emotional eating/binge eating as evidence clearly shows that it impacts on outcomes [8] but further research is needed to clarify whether it is more efficacious to provide this before or after surgery [30].

### Limitations

The aim of this survey was to document the range of psychological input across a bariatric surgery pathway rather than focusing on the technical aspects of this input (e.g. methods, mode, intensity etc.). However, in order to make the most meaningful interpretations it would be helpful to gather detailed data about the amounts of psychological input provided before and after surgery in order to optimise comparison and make meaningful conclusions about the likely impact on patient care. Whilst this survey provides data on how many of the psychologists offer input at various junctures in the pathway, we do not know how many patients actually receive this input. A further limitation of this study is that it is not possible to calculate a precise response rate. The survey was distributed via an online forum and the members consist of a mix of psychologists working in bariatric surgery or weight management services and the precise membership numbers of each group is unknown. It is equally difficult to obtain accurate information about the number of NHS bariatric units in the UK as submission to the National Bariatric Surgery Register is voluntary. In order to address these shortcomings, we used data on the total number of bariatric procedures carried out by the NHS in the same year that this survey was undertaken and calculated that the total number of procedures performed by the services included in this sample accounted for a significant proportion of these.

## Conclusion

Bariatric surgery involves a psychologically vulnerable client group undergoing a psychologically challenging and demanding procedure that is dependent on significant behaviour change and the development of new coping skills. This survey indicates that there is significant variation in the provision and scope of NHS bariatric psychology services in the UK. There is a need for a clear and consistent relationship between the amount of psychology resources and surgery volume. In addition, guidelines are needed regarding the minimum (and optimal) level of psychological input throughout a bariatric surgery pathway. It is striking that most of the psychological input seems to be directed at pre-operative assessment yet delivering pre and post-operative interventions is likely to have more impact on outcomes. Given the prevalence of mental health problems amongst this population, psychological complications should be expected and having the capacity, resources and skills to respond to these is critical. In order to make optimum use of psychologist’s skills, we need to configure bariatric psychology services to provide pre-surgery interventions focused on improving readiness and preparation for surgery, and post-surgery interventions which counteract emerging difficulties which could jeopardise weight loss and psychosocial outcomes.
